# Supplementary material for: Studies of global and local entanglements of individual protein chains using the concept of knotoids
Source: Sci Rep. 2017 Jul 24;7:6309. doi: 10.1038/s41598-017-06649-3 (PMC5524787; doi:10.1038/s41598-017-06649-3)
Supplement: Supplementary file 1 — Supplementary Information [file 41598_2017_6649_MOESM1_ESM.pdf]

Supplementary information
Studies of global and local entanglements of individual protein chains using the concept of knotoids

Dimos Goundaroulis[1,2], Julien Dorier[1,3], Fabrizio Benedetti[1,3], and Andrzej Stasiak[1,2]

[1]Center for Integrative Genomics, University of Lausanne, 1015 Lausanne, Switzerland
[2]Swiss Institute of Bioinformatics, 1015 Lausanne, Switzerland
[3]Vital-IT, SIB Swiss Institute of Bioinformatics, 1015 Lausanne, Switzerland

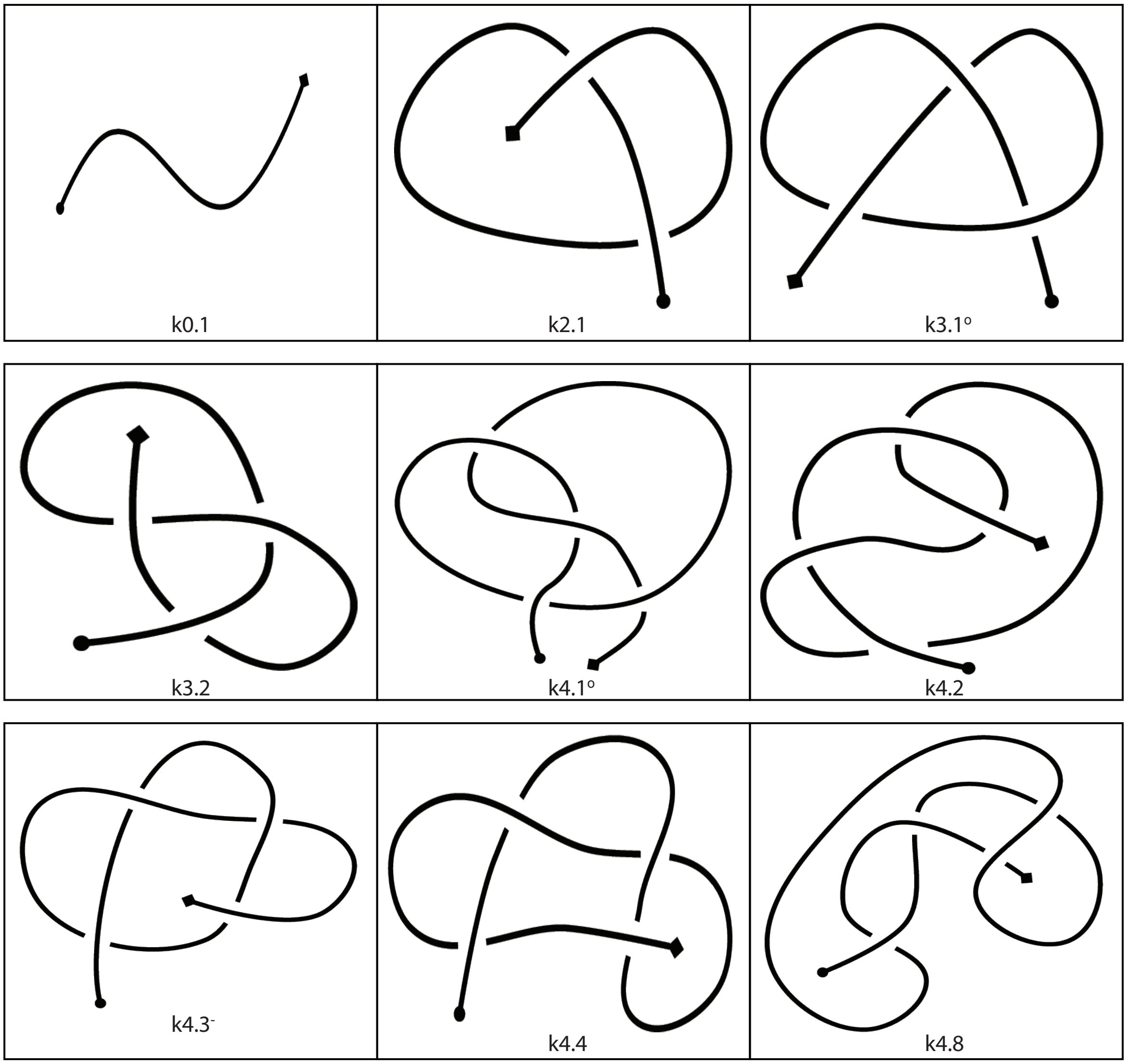

Figure S1. Knotoid diagrams that appear in the present study.

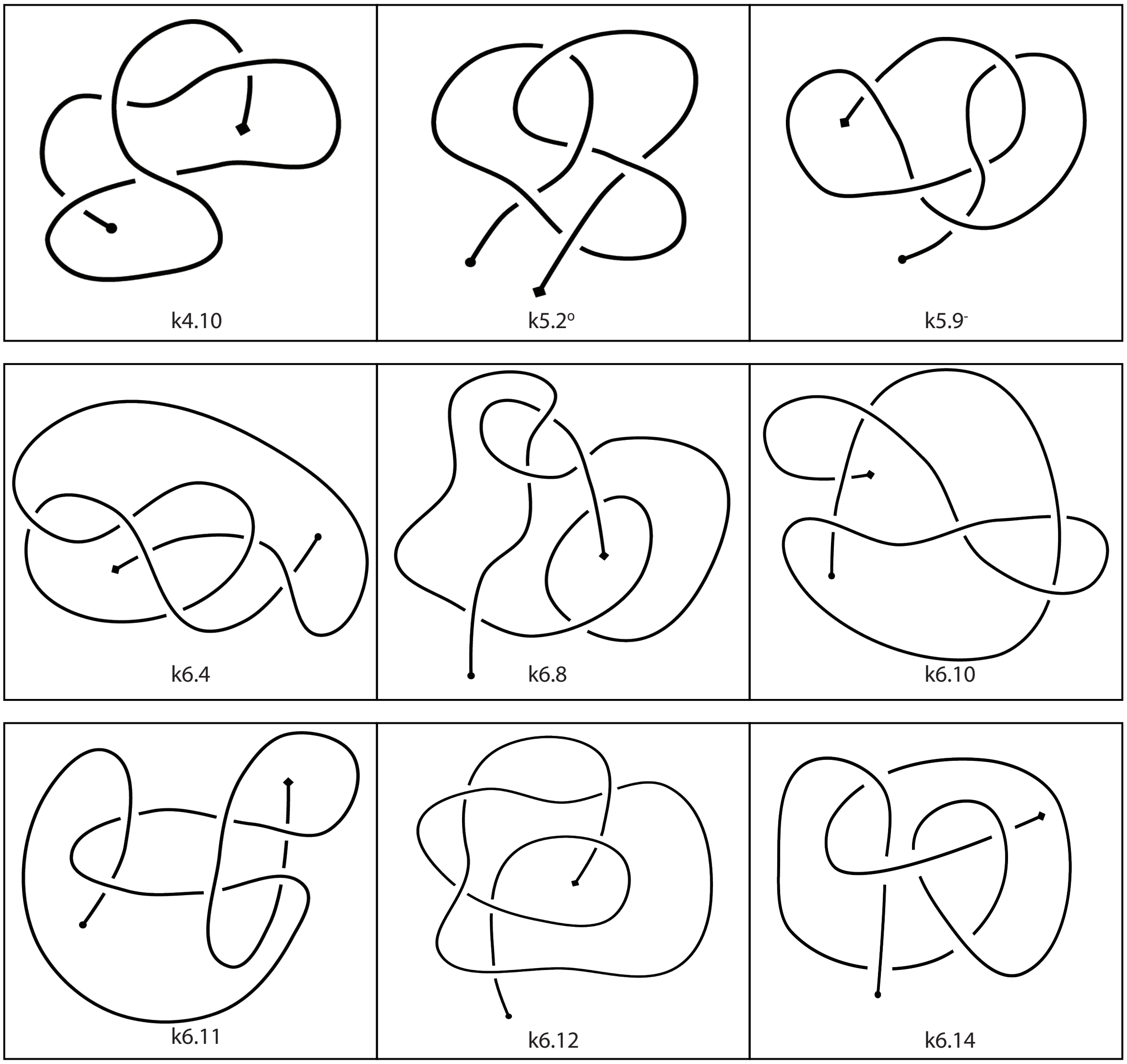

Figure S2. Knotoid diagrams that appear in the present study.

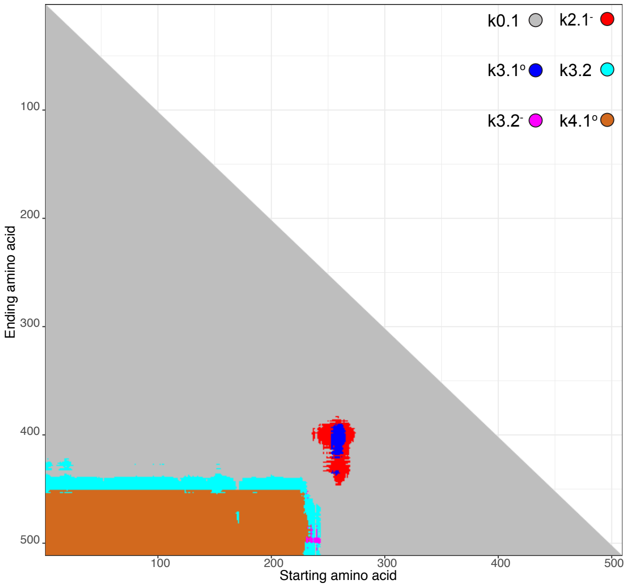

(a) 1YVE

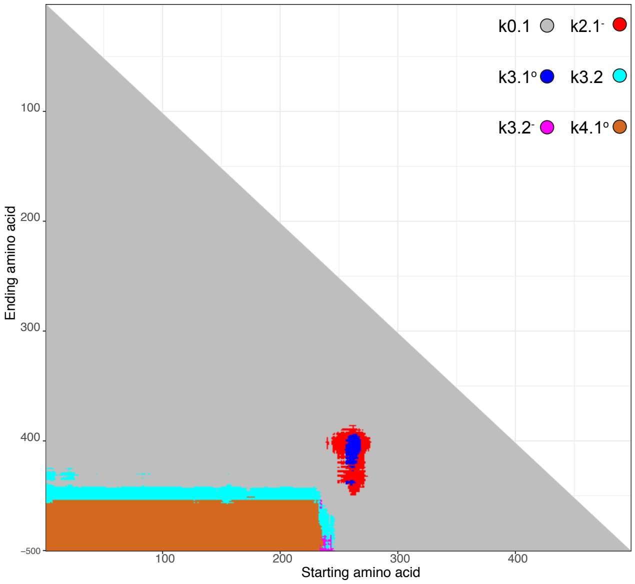

(b) 3FR8

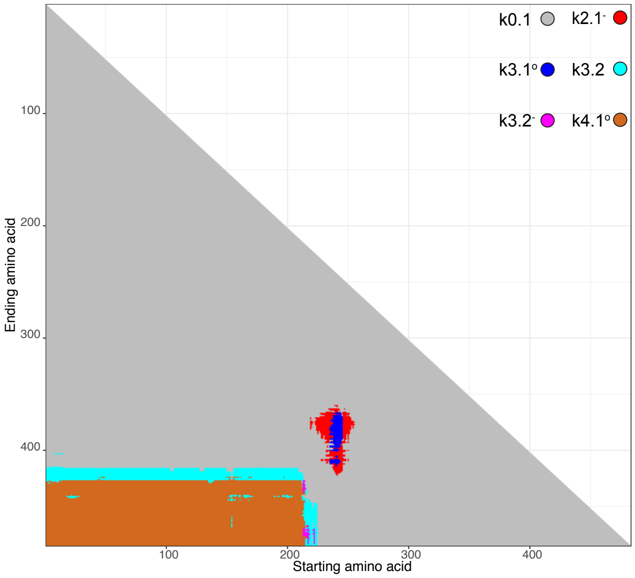

(c) 1YRL

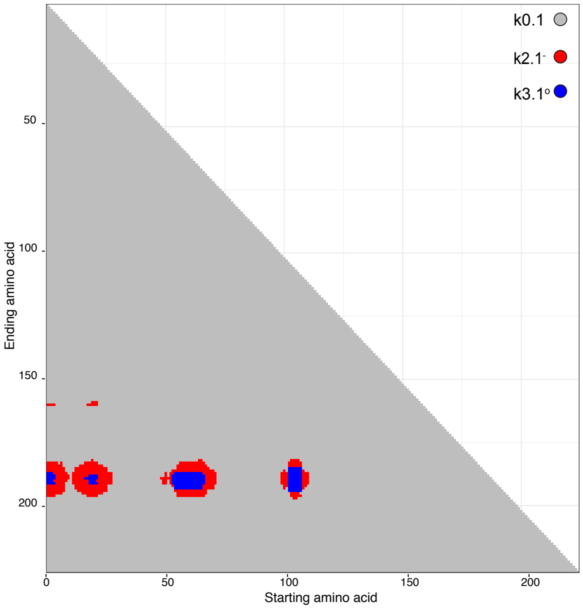

(d) 2AXC

Figure S3. Knotoid fingerprints of protein chains 1YVE, 3FR8, 1YRL and 2AXC.

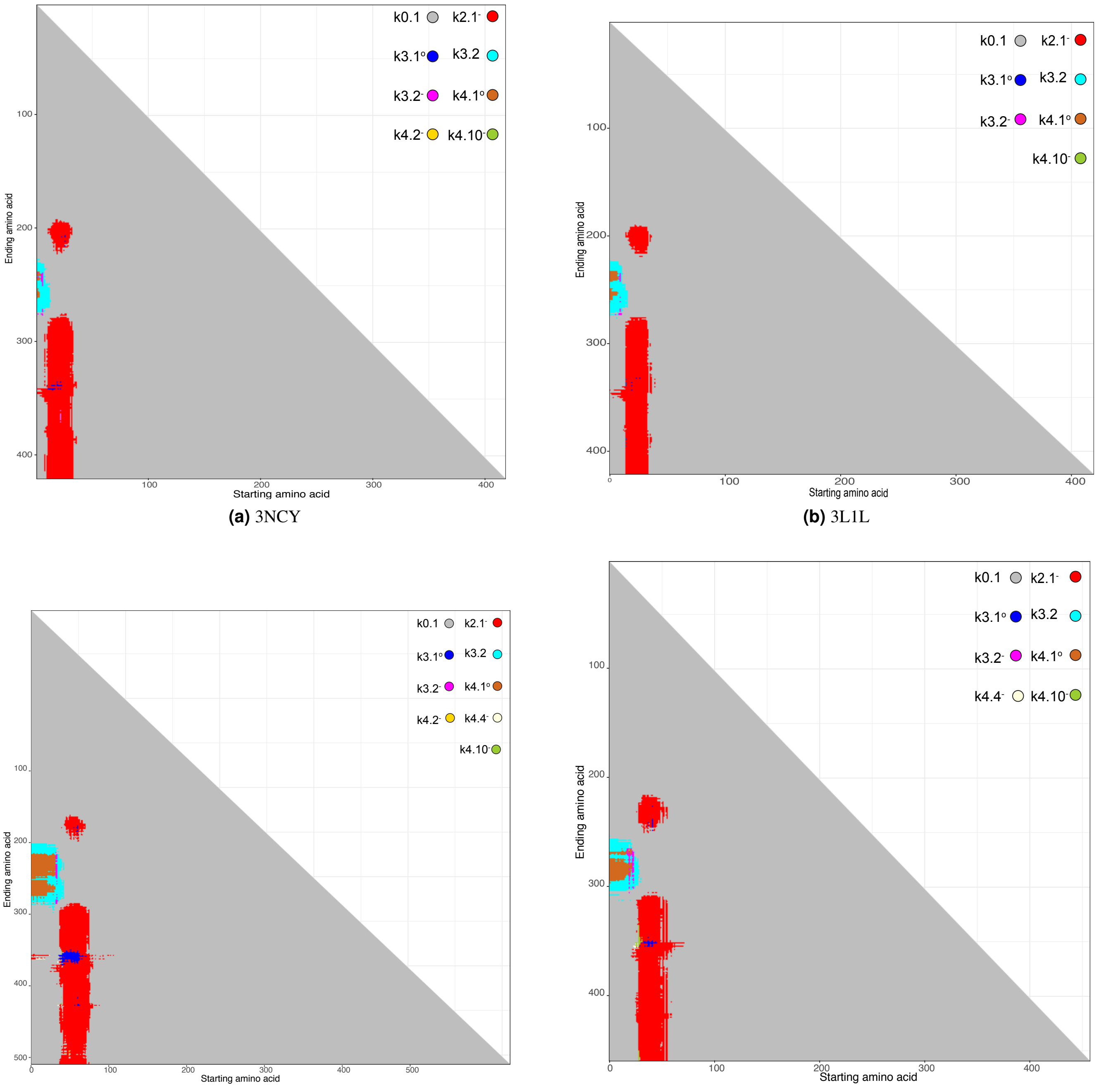

(a) 3NCY **(b)** 3L1L **(c)** 3DH4 **(d)** 2JLO

Figure S4. Knotoid fingerprints of protein chains 3NCY, 3L1L, 3DH4 and 2JLO.

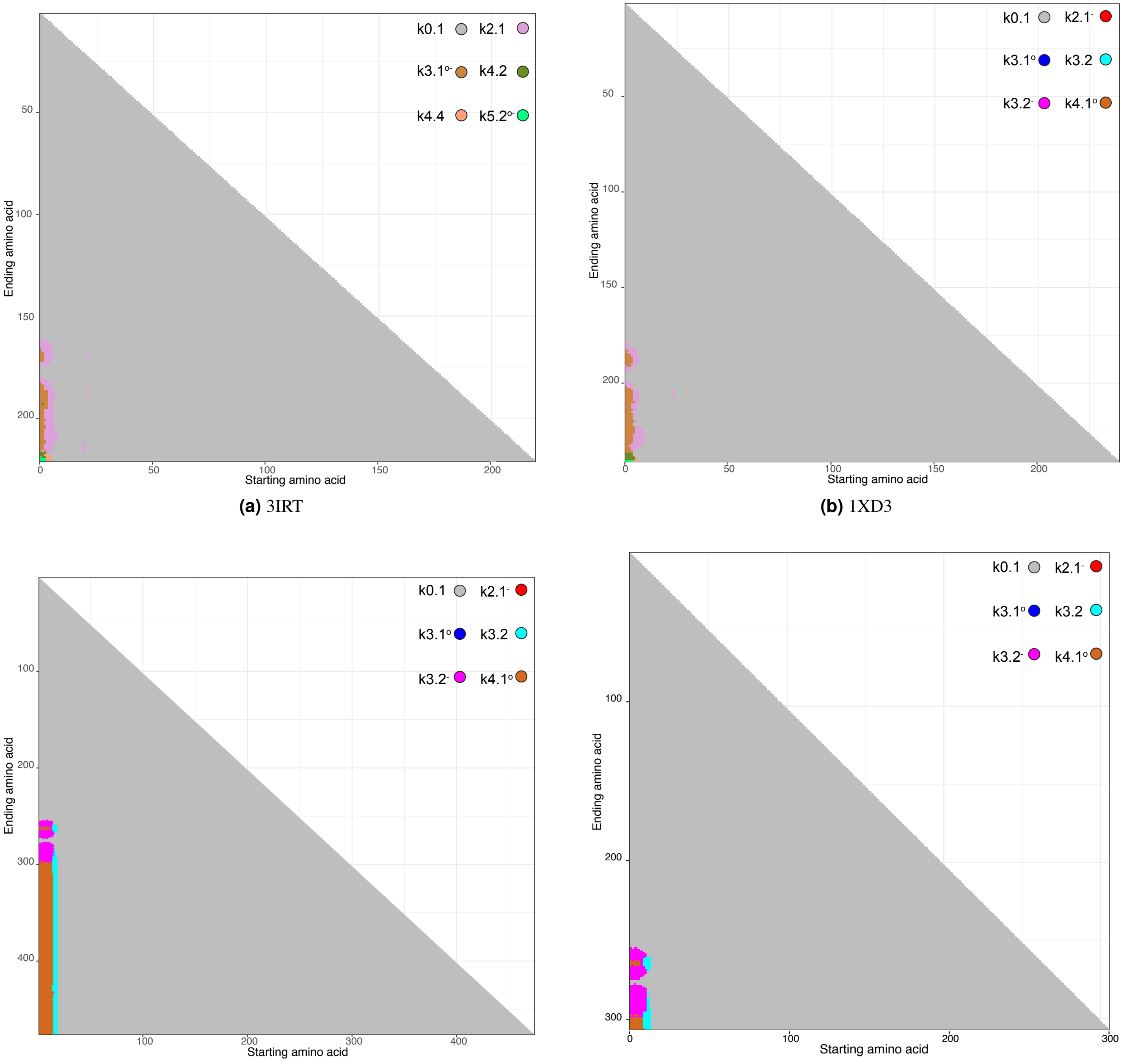

(a) 3IRT

(b) 1XD3

(c) 3C2W

(d) 2OOL

Figure S5. Knotoid fingerprints of protein chains 3IRT, 1XD3, 3C2W and 2OOL.
